# The development of an environmental risk score using Swedish National Registers and its impact on subsequent episodes of major depression

**DOI:** 10.1017/S0033291725000583

**Published:** 2025-03-07

**Authors:** Kenneth S. Kendler, Sara L. Lönn, Jan Sundquist, Kristina Sundquist

**Affiliations:** 1 Virginia Institute for Psychiatric and Behavioral Genetics, Virginia Commonwealth University, Richmond, VA, USA; 2 Department of Psychiatry, Virginia Commonwealth University, Richmond, VA, USA; 3 Center for Primary Health Care Research, Lund University, Malmö, Sweden; 4 University Clinic Primary Care Skåne, Region Skåne, Sweden

**Keywords:** major depression, environmental risk scores, genetic risk, stressful life events, co-sibling control

## Abstract

**Background:**

Stressful life events (SLEs) increase the risk for subsequent major depression (MD) episodes. Most prior studies of SLEs utilized questionnaires or interview-based assessments. We sought to develop and evaluate an environmental risk score (ERS) for MD from multiple classes of SLEs obtained from national Swedish registries.

**Methods:**

We assessed, in the entire adult population of Sweden (*n* = 7,105,712), the occurrence of 52 categories of SLEs derived from registry information for the 6 months prior to January 9, 2010 and the risk for MD registration over the subsequent 6 months. Weights for our ERS were obtained from a random half of our sample and ERS and its relationship to MD risk was evaluated in the second half.

**Results:**

The ERS was robustly related to risk for subsequent MD episodes. Women were more sensitive to the depressogenic effect of the ERS than men. Those with prior episodes of MD had larger absolute increases in MD risk from our ERS than those without previous episodes. Genetic risk for MD was associated with a greater sensitivity to the depressogenic effects of the ERS. A co-sibling control analysis suggested that most, but not all, of the association between ERS and subsequent risk for MD was causal.

**Conclusions:**

Valid measures of SLEs that predispose to risk for MD can be assessed from high-quality registry data. While not all event categories (e.g. interpersonal or romantic difficulties) can be assessed, this method avoids problems with accurate dating and recall bias and can be performed in very large samples.

## Introduction

A long research tradition has examined the association between stressful life events (SLE) and risk for episode onset or recurrence of psychiatric disorders, particularly major depression (MD) (Brown & Harris, 1978; Brown, Harris, & Hepworth, 1995; Cohen, Murphy, & Prather, 2019; Holmes & Rahe, 1967; Kendler, Karkowski, & Prescott, 1998; Kessler, 1997; Paykel et al., 1969). A substantial proportion of this association appears to be causal (Kendler & C.O. Gardner, 2010a; Kendler, Karkowski, & Prescott, 1999). Nearly all such studies have relied on a questionnaire or interview-based retrospective assessments of SLEs completed by respondents with the interview-based measures likely producing more valid results (Brown, Sklair, Harris, & Birley, 1973; Dohrenwend, Link, Kern, Shrout, & Markowitz, 1987; Dohrenwend, Raphael, Schwartz, & Skodol, 1993; Dohrenwend, Dohrenwend, Dodson, & Shrout, 1984; Paykel, 1983). However, such assessments are time-consuming and typically available on only limited samples. Furthermore, problems of interpretation remain including accurate timing of events and memory biases and/or forgetting (Brown et al., 1973; Raphael & Cloitre, 1994; Raphael, Cloitre, & Dohrenwend, 1991), which would not impact on SLEs that were assessed from objective sources.

To begin to address these concerns, in a prior publication, we explored the utilization of one specific and severe SLE assessed from Swedish registry information and showed that, consistent with a range of prior studies based on interview-assess SLEs (Brown et al., 1995; Kendler et al., 1998), the death of a close relatives (parents, siblings, spouse, and children) was associated with a substantial and sustained increased risk for incident and recurrent episodes of MD as well as for stress reaction and alcohol use disorder (Kendler, Lonn, Sundquist, & Sundquist, 2023). In this report, we describe our efforts to create an environmental risk score (ERS) utilizing the wide variety of kinds of adversities that can be assessed using the Swedish national registries which sums the events weighted by their predictive impact on MD risk. Conceptually, we took this approach with the goal of developing a rough environmental proxy to the now commonly used measures of aggregate genetic risks such as polygenic risk scores (PRS) and the family genetic risk score (FGRS).

Then we evaluate this ERS in five specific ways. First, do we see the expected dose-response relationship seen in prior studies utilizing interview-based assessments of SLEs (Frank et al., 1996; Kendler et al., 1998) – in which multiple SLEs, reflected in our analyses as a higher ERS score – more strongly predicts risk for MD? Second, do we see differences across the sexes in their overall sensitivity to the depressogenic effect of our ERS (Assari & Lankarani, 2016; Kendler, Thornton, & Prescott, 2001; Maciejewski, Prigerson, & Mazure, 2001)? Third, does our ERS score interact with a prior history of MD in predicting depressive episodes? Fourth, do we see a positive interaction between our ERS and the genetic risk for MD – as assessed by our FGRS – in the prediction of MD (Kendler et al., 1995; Tennant, 2002)? Finally, we attempt to assess the causal nature of the ERS-MD association by utilizing a co-sibling design (Kendler et al., 1999).

## Methods

### Data sources

We linked several Swedish Nationwide registers using the unique 10-digit identification number assigned at birth or immigration to all Swedish residents. The identification number was replaced by a serial number to ensure people’s privacy. The sources for our dataset are seen in Supplementary Table 1.

### Sample and measure

We used the whole Swedish population over the age of 20 on September 1, 2010. We set the baseline to October 1, 2010, and assessed possible environmental risk factors up to 6 months before baseline and the outcome during the 6 months following baseline. The outcome, MD, was defined from medical records using the in-patient and specialist registries and the primary care data using the ICD-10 codes: F32 and F33. The possible environmental risk factors were defined from three different categories: events or diagnosis in the individual, death of a first-degree relative, and severe diagnosis in a first-degree family member. For a complete list of the 24 categories of ICD codes covering events or diagnoses in the individual, see Supplementary Table 2. We also included 10 events in first-degree family members (including biological or adoptive parents, full siblings, biological children, and spouses) distinguishing between death due to suicide and death due to other causes (see definitions in Supplementary Table 3). Seven categories of stressors, including medical conditions or criminal conviction, in biological children were included and are listed in Supplementary Table 4.

Prior MD was defined as prior to baseline and we included our previously described individual Familial Genetic Risk Scores (FGRSs) for MD. Similar to prior studies (e.g. Kendler, Ohlsson, Bacanu, Sundquist, & Sundquist, 2023; Kendler, Ohlsson, Moscicki, et al., 2023; Kendler, Ohlsson, Sundquist, & Sundquist, 2021, 2023, 2024), the FGRS were based on 1st to 5th degree relatives to the probands with a mean of 40.1 relatives per proband. To calculate the MD fgrs we require that the proband and the proband’s parents are born in Sweden. Briefly, they are calculated from morbidity risks for disorders relatives, controlling for cohabitation effects, and thus arise from phenotypes in extended pedigrees, not from molecular genetic data. They are standardized by year of birth and county of residence into a z-score with mean = 0 and SD = 1.

The first step was to develop an MD ERS by creating an aggregated measure of the possible environmental risk factors defined above. We split our entire sample into two random halves and used one half as a training set to develop our MD ERS and then we applied the risk model on the testing half to evaluate the derived MD ERS. Because we wanted to develop an MD ERS that represents an aggregate sum of risks for numerous different life events, we utilized a linear probability model with MD within 6 months as the outcome and each of the potentially SLE in the last 6 months as predictors. We thereby obtained an estimate of the weights for each of the events. The estimated parameters from the linear probability model represented the weights for the events during the defined time-periods. We applied these weights to the observed events in the testing data set and thereby obtained an MD ERS that represents the risk of MD within 6 months.

### Statistical analyses

To evaluate the MD ERS on the testing data set we first ran a linear probability model with the derived MD ERS as predictor and MD within 6 months as outcome. Theoretically, if the MD ERS perfectly matches the risk of MD within 6 months the parameter estimates of the MD ERS variable would equal one. First, we ran a series of models to evaluate the MD ERS in its prediction of MD episodes, beginning with a linear term and then, to explore a possible attenuating association for higher values of the ERS, we added a quadratic term to the linear probability model. The two models; only linear or linear and quadratic terms were compared using adjuster *R*^2^, Akaike’s information criterion (AIC) (Akaike, 1987), and a graphical evaluation, comparing the estimated regression lines to observed values.

Second, to investigate the confounding and effect modification of sex we first ran a model with only sex (Model 2b), in a second step we included both the MD ERS and sex (Model 2c) and finally to investigate if the association with MD ERS is modified by sex, we expanded Model 2c to include the interaction between MD ERS and sex (Model 2d). Third, we ran the corresponding three models including prior MD (Model 3b–d). Fourth, to explore the impact of genetic risk we ran the corresponding three models including the MD fgrs. These latter analyses were run on the subset of the population with a valid assessment of MD fgrs. Because we used linear probability models, all interactions were assessed on an additive scale.

Fifth, to account for familial confounding, we evaluated our MD ERS using a co-sibling analysis. Full sibling pairs were included in a linear mixed model, where a random effect accounted for the within-pair clustering. Pairs discordant on the level of MD ERS and MD within 6 months are informative for the estimate of the association between the MD ERS and consecutive MD. This analysis was repeated on a modified MD ERS which excluded events in family members (parents, siblings, spouses, and children). Because the MD ERS and MD fgrs are on different scales the parameter estimated of the two variables cannot be compared and we instead present the results as figures.

## Results

### Descriptive results

Our main descriptive results are presented in Table 1. Our entire sample included 7,105,712 individuals of whom 11.3% had at least one SLE in the 6 months prior to January 9, 2010 and 2.0% had an MD registration over the 6 months after January 9, 2010. Compared to the general population, the individuals with MD episodes both had a higher proportion of SLEs (16.9% vs. 11.3% in the last 6 months) and had a considerably higher level of FGRS_MD_ (0.33 vs. −0.01). We then subdivided our main sample by sex, presence or absence of a prior MD episode, and age. As expected, rates of MD in the 6 months after January 9, 2010 were approximately twice as high in women as in men.

**Table 1.** Descriptive statistics for the relationship, in our population cohort, between any stressful life events in the 6 months prior to September 1, 2010 and episodes of major depression in the 6 months after^*^

	In total (col %)	MD within 6 months (col %)	In total (col %)	MD within 6 months (col %)
Whole sample	7,105,712	139,318 (1.96%)		
SLE last 6 months	802,240 (11.29%)	23,498 (16.87%)		
Age, mean (SD)	56.1 (19.93)	56.25 (18.62)		
MD fgrs, mean (SD)	−0.01 (0.99)	0.33 (1.14)		
By gender	Males		Females	
All	3,517,431	46,674 (1.33%)	3,588,281	92,644 (2.58%)
SLE last 6 months	386,909 (11.00%)	7913 (16.95%)	415,331 (11.57%)	15,585 (16.82%)
Age, mean (SD)	55.28 (19.79)	55.33 (18.01)	56.92 (20.03)	56.71 (18.9)
MD fgrs, mean (SD)	−0.02 (0.99)	0.32 (1.14)	0 (1)	0.34 (1.14)
By prior MD status	No prior MD episode		At least one prior MD episode	
All	6,542,809	45,506 (0.70%)	562,903	93,812 (16.67%)
SLE last 6 months	714,879 (10.93%)	7414 (16.29%)	87,361 (15.52%)	16,084 (17.14%)
Age, mean (SD)	56.07 (20.06)	55.67 (19.83)	56.46 (18.39)	56.53 (18.00)
MD fgrs, mean (SD)	−0.03 (0.98)	0.26 (1.11)	0.31 (1.12)	0.37 (1.15)
By prior MD status	Younger than 50		Older than 50	
All	3,088,426	58,917 (1.91%)	4,017,286	80,401 (2.00%)
SLE last 6 months	294,581 (9.54%)	8666 (14.71%)	507,659 (12.64%)	14,832 (18.45%)
Age, mean (SD)	37.79 (7.54)	38.65 (7.36)	70.19 (14.24)	69.15 (12.94)
MD fgrs, mean (SD)	0.00 (1)	0.40 (1.15)	−0.01 (0.99)	0.29 (1.12)

* Abbreviations: FGRS, family genetic risk score; MD, major depression; SLE, stressful life events.

**Figure 1.**
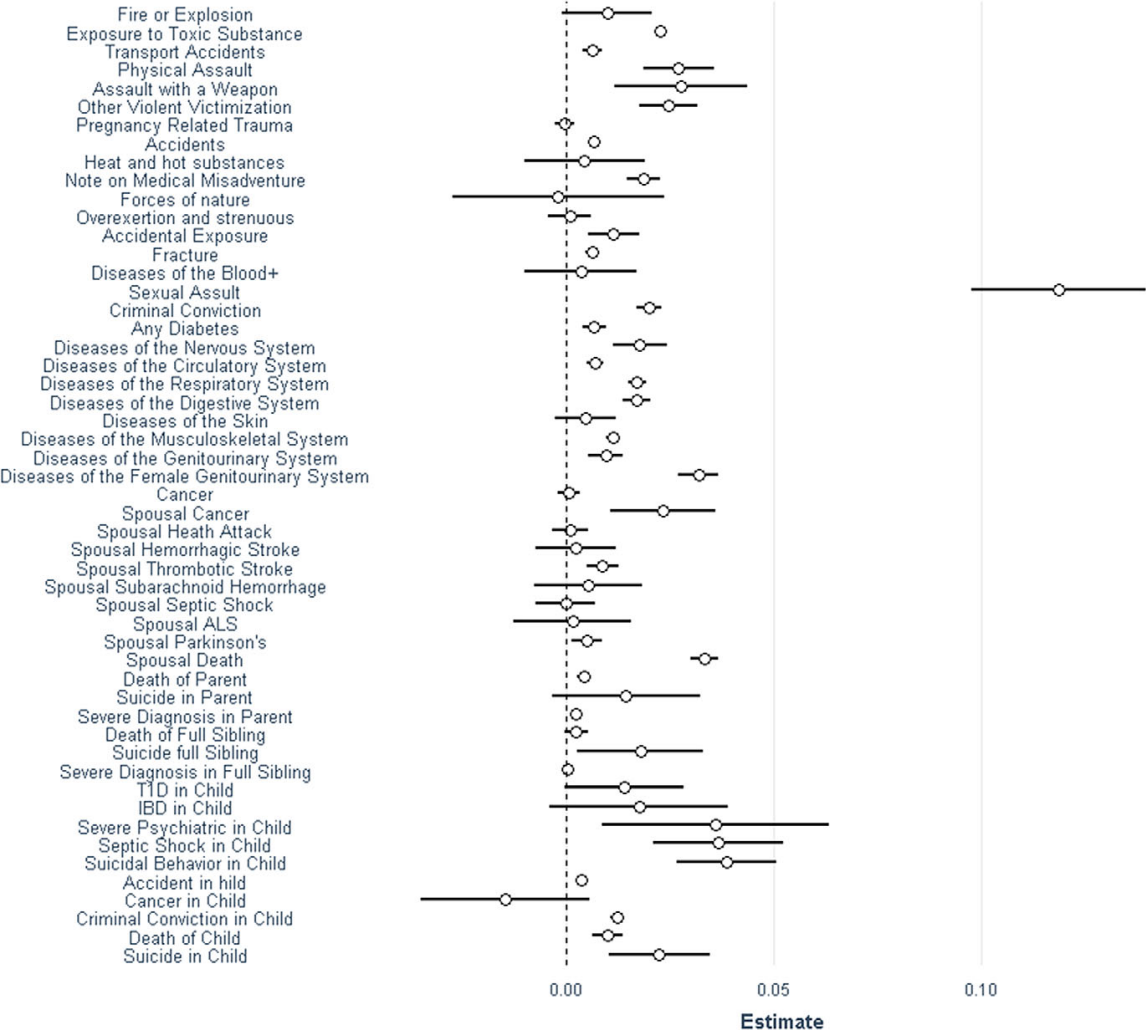
The individual stressful life events contributing to our environmental risk score. See Supplementary Table 2 for the ICD codes corresponding to these individual events. The *X*-axis represents the contribution of the individual stressful event to the ERS (±95% CIs).

We examined the association between each of our 52 SLEs when they occurred within 6 months of January 9, 2010 and the presence of a depressive episode during the 6 months after that date in Figure 1. The highest impact event on MD risk was sexual assault with other SLEs with substantial predictive power including assault with a weapon, a criminal conviction, psychiatric illness and suicidal behavior in a child, spousal cancer, and death and parental death. More moderate risks were seen with events such as a range of medical diagnoses, accidents, fractures, and diabetes or criminal convictions in a child.

### Analysis of our ERS

**Figure 2.**
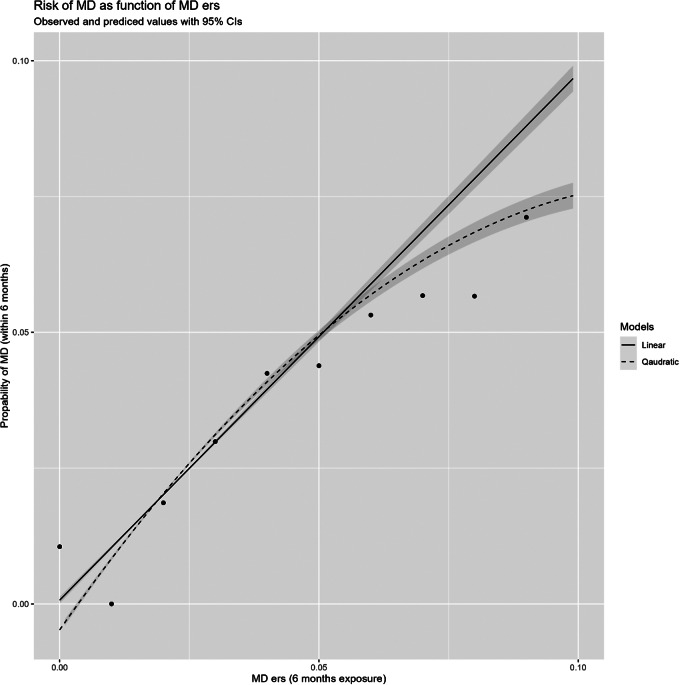
The risk for major depression as a function of the environmental risk score (ERS) within 6 months of the exposure window. The points are observed findings to which we fit both a linear and a linear + quadratic model. The *Y*-axis is the probability of MD and the *X*-axis is the aggregate ERS score.

We first describe the overall univariable relationship, including 95% CIs, between our ERS score over the 6-month period prior to January 9, 2010 with the risk for MD in the 6 months after that date, in Figure 1. A linear model fitted the data well but the model was modestly improved by the addition of a quadratic function with the fit dropping 88 AIC units and a modest improvement in predicted variance. However, for simplicity, our further modeling efforts utilized the linear model (see Figure 2).

**Figure 3.**
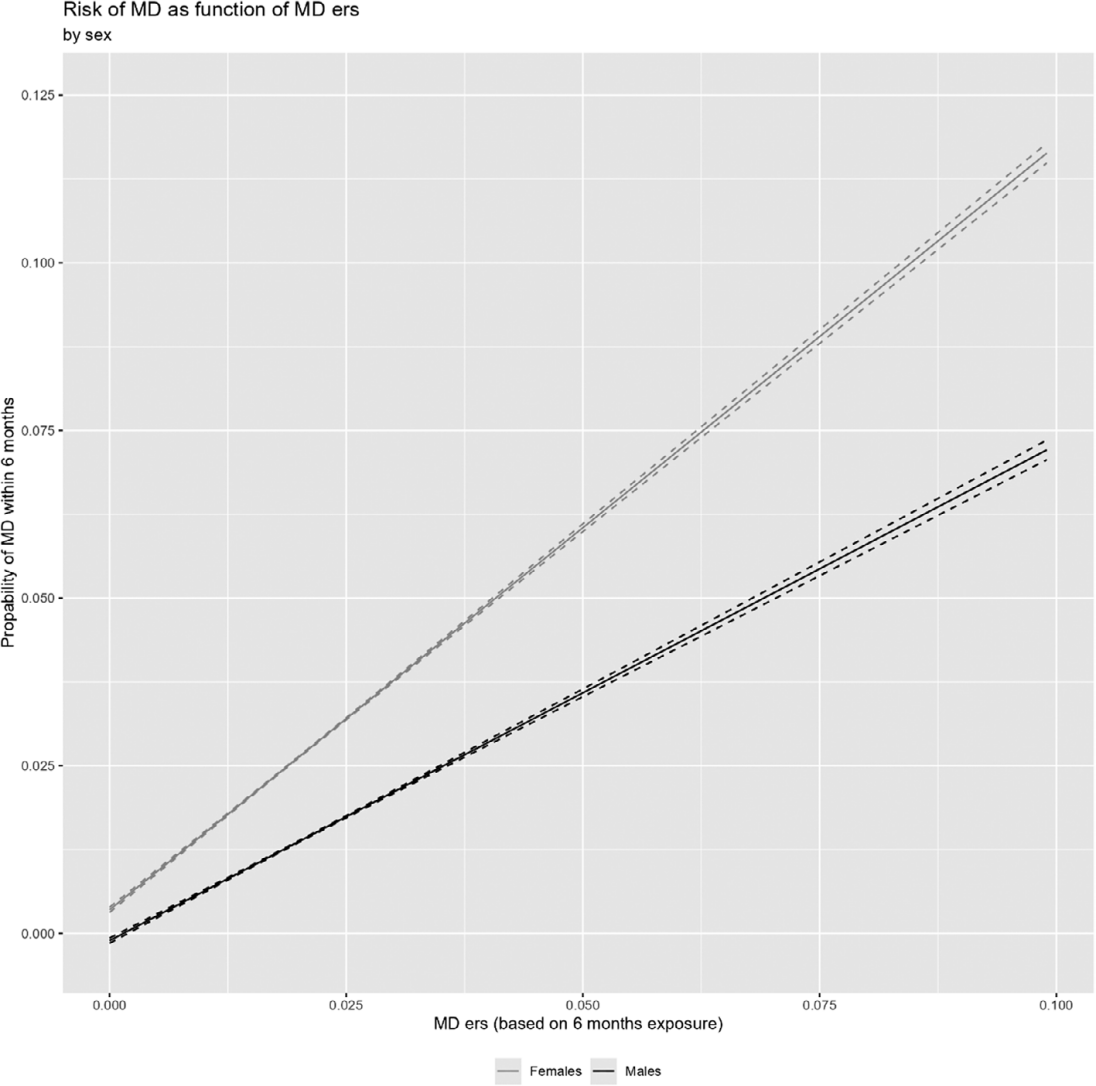
The risk for major depression as a function of the environmental risk score (ERS) within 6 months of the exposure window in men and women. The *Y*-axis is the probability of MD and the *X*-axis is the aggregate ERS score.

**Figure 4.**
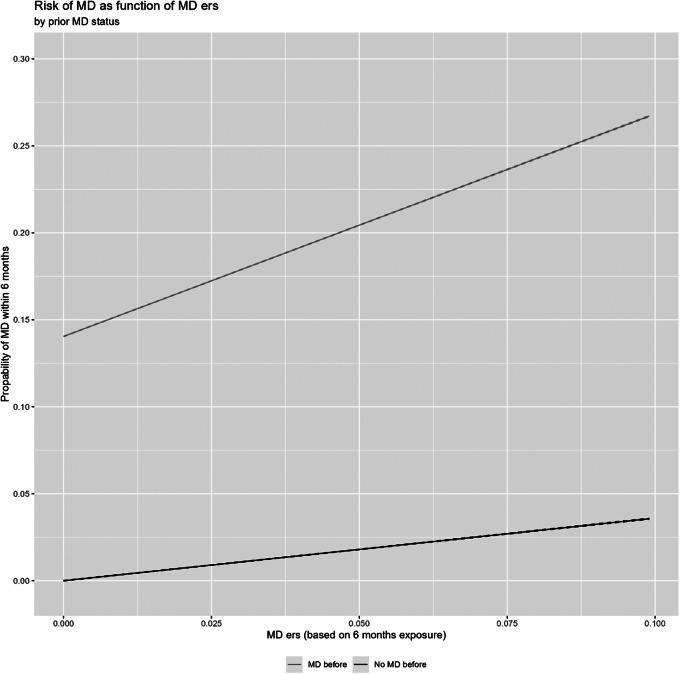
The risk for major depression as a function of the environmental risk score (ERS) within 6 months of the exposure window in the presence or absence of a prior episode of major depression. The *Y*-axis is the probability of MD and the *X*-axis is the aggregate ERS score.

**Figure 5.**
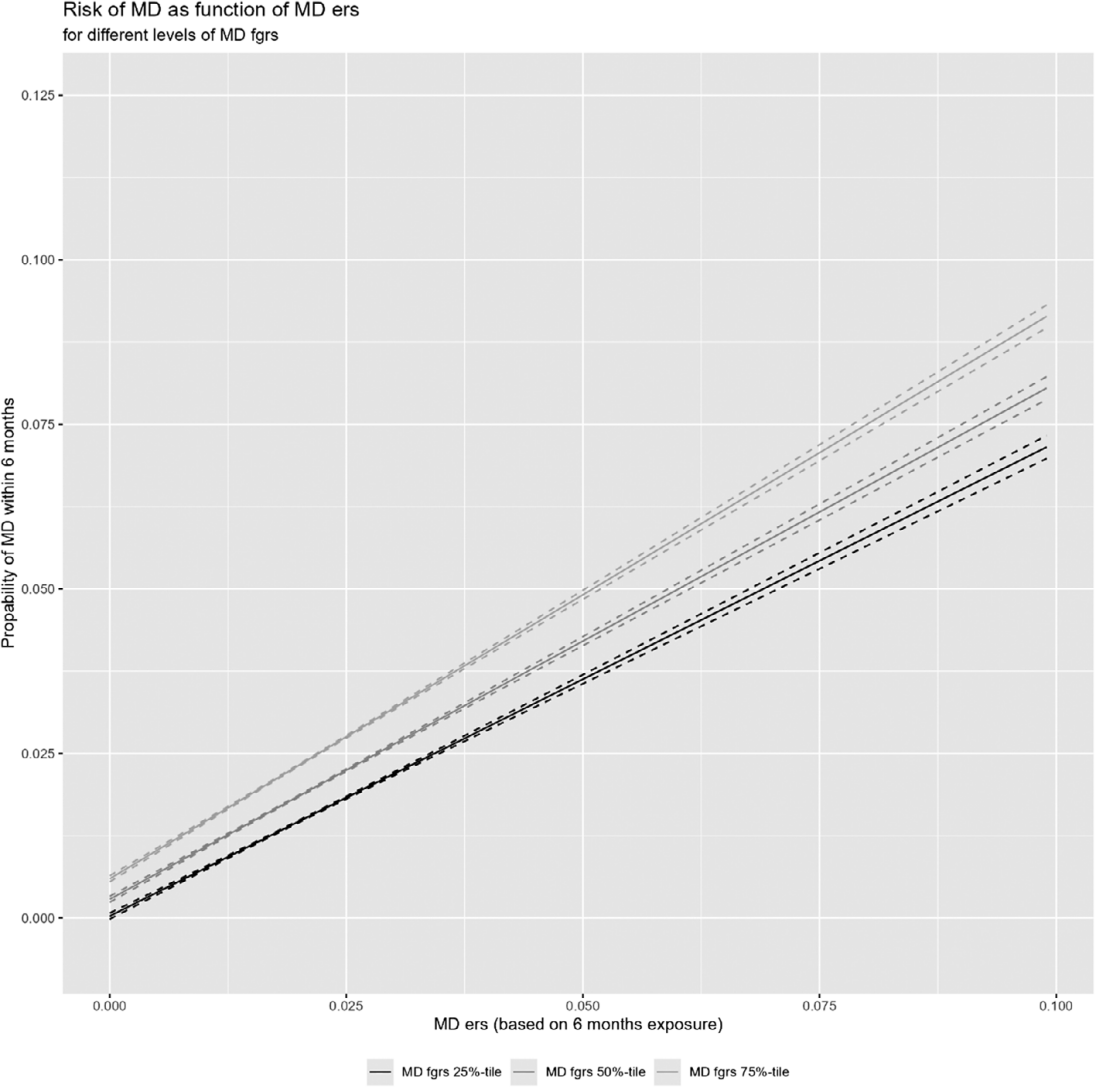
The risk for major depression as a function of the environmental risk score (ERS) within 6 months of the exposure window as a function of the level of genetic risk for major depression as assessed by the level of the FGRS_MD_ depicted at the 25th, 50th, and 75th percentile. The *Y*-axis is the probability of MD and the *X*-axis is the aggregate ERS score.

We then fitted a series of three more complex models with the results presented in Table 2 and Figures 3–5. The first examines, in Table 2 part 1, ERS, sex, and their interaction in the prediction of subsequent MD episodes. Models A and B provide the univariate impact on MD risk for, respectively, ERS and sex both of which are highly significant. Model D presents the multivariate model containing the two predictors and model D presents these two main effects and their interaction, which is also highly statistically significant. The results are presented in Figure 3 and illustrate that women are significantly more “sensitive” to the depressogenic effects of our ERS than men. To clarify whether this overall effect results from a few events to which women were much more sensitive or a more general higher sensitivity in women across many event categories, we examined sex differences in response to all of our events (Supplementary Figure 1). Consistent with the second hypothesis, for a substantial proportion of our SLEs, the impact of event exposure predicted MD more strongly in women than in men.

**Table 2.** Results of analyses of the prediction of major depression by the environmental risk score (ERS) and Sex, ERS and Prior MD and ERS and genetic risk for MD^a^

Model	Predictor variables	Specific regression model with beta estimate and 95% CIs			
		Models A	Models B	Models C	Models D
ERS, Sex and their interaction	Intercept	0.0007 (0.0001, 0.0013)	0.0133 (0.0131, 0.0135)	−0.0054 (−0.006, −0.0047)	−0.0011 (−0.0020, −0.0002)
	MD ers	0.9698 (0.9400, 0.9997)	–	0.9589 (0.9290, 0.9887)	0.7392 (0.6948, 0.7837)
	Female		0.0125 (0.0122, 0.0128)	0.0124 (0.0121, 0.0127)	0.0046 (0.0034, 0.0058)
	Interaction MD ers × Female				0.4004 (0.3405, 0.4604)
	Adjusted *R*-squared	0.0011	0.0020	0.0031	0.0032
ERS, Prior MD episode and their interaction	Intercept	0.0007 (0.0001, 0.0013)	0.0070 (0.0068, 0.0071)	−0.0028 (−0.0034, −0.0023)	0.000 (−0.0006, 0.0006)
	MD ers	0.9698 (0.9400, 0.9997)		0.5042 (0.4757, 0.5326)	0.3602 (0.3292, 0.3912)
	Prior MD episode		0.1595 (0.1590, 0.1600)	0.1590 (0.1585, 0.1595)	0.1405 (0.1388, 0.1421)
	Interaction MD ers × prior MD episode				0.9190 (0.8408, 0.9973)
	Adjusted *R*-squared	0.0011	0.0966	0.0969	0.0970
ERS, FGRS_MD_ and their interaction^b^	Intercept	0.0027 (0.002, 0.0034)	0.0195 (0.0193, 0.0197)	0.0035 (0.0028, 0.0042)	0.0037 (0.0030, 0.0044)
	MD ers	0.8567 (0.822, 0.8914)	–	0.8209 (0.7862, 0.8556)	0.8073 (0.7723, 0.8422)
	MD fgrs		0.0067 (0.0065, 0.0068)	0.0066 (0.0064, 0.0067)	0.0044 (0.0037, 0.0051)
	Interaction MD ers × MD fgrs				0.1104 (0.0779, 0.1429)
	Adjusted *R*-squared	0.0009	0.0023	0.0031	0.0032

a FGRS_MD_, family genetic risk score for MD.

b This model was run on the subset of the sample that had valid MD FGRS measures.

Figure 4 (and Table 2 part 2) then examines the impact of prior MD on the ERS-MD relationship. We can see both from the table and figure, that from the perspective of an additive model, the increase in rates of MD for those with a prior MD upon ERS exposure is greater than that seen with those with no prior episode.

Finally, with Figure 5 (and Table 2 part 3), we address the question of Gx E effects. We depict in this figure, the risk for MD across the range of our ERS scores in those with FGRS_MD_ scores in the 25th, 50th, and 75th percentile. The slope is modestly steeper consistent with the significant positive interaction seen between ERS and FGRS in Table 2 in the prediction of MD.

### Co-sibling analyses

Given that a range of confounders could both influence the risk of SLEs and depressive episodes, it would be inappropriate to assume that the predictive associations that we describe between SLE exposure and subsequent risk for MD are entirely causal. To give us some insight into the role of familial confounders (that is those factors which tend to run in families and are associated both with the experience of SLEs and episodes of MD) that impact the SLE-MD associations that we describe, we conducted co-sibling control analyses. We compared the beta estimate in our entire population for the ERS-MD relationship noted above, which was calculated at +0.97 (0.94; 1.00) to that obtained within pairs of full siblings which equaled +0.82 (0.78–0.86). These results are consistent with a modest degree of familial confounding. We then repeated the analyses removing from our ERS all of the events that involve family members and those would likely be shared among siblings. The results changed little equaling +0.84 (0.80–0.89).

## Discussion

We sought, in this report, to evaluate the performance of our ERS, created from national medical, criminal, and death registry data in Sweden, in the prediction of risk for MD. Of our many findings, we consider six to be of particular importance and review them here.

First, a review of the individual events, derived from Swedish national registries, that were most strongly related to risk for MD – including sexual and physical assault, assault with a weapon, spousal cancer and death and severe psychiatric illness in a child – are both clinically face-valid and agree with other empirical studies of the kinds of events that are particularly depressogenic (Brown & Harris, 1978; Finlay-Jones & Brown, 1981; Kendler, Hettema, Butera, Gardner, & Prescott, 2003; Kendler, Thornton, & Gardner, 2001).

Second, higher scores on our ERS were associated in a largely linear manner, with increasing risk for MD, consistent with prior findings that severe events and/or multiple stressful events occurring close together in time have an increased aggregate effect of depressive risk (Frank et al., 1996; Kendler et al., 1999; Miller & Ingham, 1985).

Third, consistent with some (Maciejewski et al., 2001), but not all prior studies, we found women to be substantially more sensitive to the depressogenic effects of our ERS than men. Prior evidence is conflicting in suggesting a greater overall sensitivity to the depressogenic effects of environmental stressors in men (Assari & Lankarani, 2016), women (Maciejewski et al., 2001) or no overall difference but greater sensitivity in the sexes to different classes of SLEs (Kendler, Thornton, & Prescott, 2001). Our results – seen in Supplementary Figure 1 – add to this literature as we show a number of specific events with considerably stronger depressogenic effects in women (e.g. assault with a weapon, criminal conviction, and spousal cancer) and far fewer events with stronger depressogenic effects in men (e.g. physical assault and spousal heart attack).

Fourth, the use of an additive model showed a greater absolute increase in risk for MD after ERS in those with versus without a prior episode of MD. It is helpful to reanalyze this data using more traditional multiplicative approaches (Supplementary Table 5). Here we show, using logistic regression, that those with a prior MD are less sensitive to the effects of environmental stressors with an HR of 0.81 per prior depressive episode, meaning an approximately 19% reduced sensitivity to stressors for each previous episode. These results are consistent with the substantial literature on the “kindling hypothesis” for MD in which, over episodes, MD becomes more autonomous and less associated with prior stressors (Kendler, Thornton, & Gardner, 2000; Monroe & Harkness, 2005; Post, 1992). However, while this data – changes in the association between SLEs and MD episodes over time – is consistent with the kindling hypothesis – but does not present any direct evidence of the specific mechanism involved.

This analysis shows the importance of the scale of measurements when assessing interactions. There is no “right” versus “wrong” way as the two scales represent different ways of conceptualizing the meaning of an interaction (Kendler & C. O. Gardner, 2010b). We favor the additive model because it takes a public health rather than a statistical view of interactions. The additive model answers the practical question of how many more cases of illness one can predict in the presence of both risk factors versus the additive effect of the two when acting alone (Kendler & C. O. Gardner, 2010b).

Fifth, several prior twin studies, assessing a range of environmental adversities, show evidence of increased sensitivity to the depressogenic effects of these environmental exposures in those with high genetic risk (Kendler et al., 1995; Strachan, Duncan, Horn, & Turkheimer, 2017; Wichers et al., 2009). Using a quite different design, we were able to replicate these findings suggesting that, with respect to MD, genes in part act on liability by altering the sensitivity to the effects of environmental stressors (Kendler et al., 1995).

Sixth, we attempted to address the difficult problem assessing the causal relationship between SLEs and MD. SLEs are modestly heritable (Bolinskey, Neale, Jacobson, Prescott, & Kendler, 2004) and/or run in families (Middeldorp, Cath, Vink, & Boomsma, 2005) and prior evidence has shown that genetic risk for MD increases exposures to certain classes of SLEs (Kendler & Karkowski-Shuman, 1997). So, it is a priori likely that the association between SLEs and MD would be at least in part mediated by familial confounders. We attempted to evaluate the presence and magnitude of this familial confounding by assessing the SLE-MD relationship using a co-sibling design. Consistent with two prior uses of this design in the assessment of the SLE-MD relationship (Bjorndal, Kendler, Reichborn-Kjennerud, & Ystrom, 2023; Kendler et al., 1999), we saw only a modest attenuation of the association. Of note, our method does not completely control for possible familial confounding because full sibs only share around 50% of their genetic risk. However, our findings do suggest that SLEs likely have a substantial causal effect on MD risk.

## Limitations

These results should be interpreted in the context of a range of potential methodological limitations. First, official Swedish registry data did not permit the assessment of all relevant environmental stressors for MD. For example, no codes for serious relationship difficulties with relatives or in the workplace, loss of confidants, and crises in one’s extended social network, all of which meaningfully increase the risk for subsequent MD episodes (Kendler et al., 1998), were available in the nationwide Swedish registries. So, our method of SLE assessment covers a more restricted range of adversities than is possible with interview or questionnaire-based assessments.

Second, in assessing, as we do here, the aggregate effects of multiple individual events, some compromises are necessary in the timing of the exposure and the possible depressive outcome. In the preliminary stages of this project, we explored 3 versus 6-month time frames for the assessment of our ERS finding the latter to have a moderately stronger predictive impact on MD risk. However, this means that we are assessing all SLEs that occurred in our sample from March 1 to August 30, 2010 and their relationship with MD episodes that occurred from September 1, 2010 until February 28, 2011. If a severe SLE occurred early in our assessment period (e.g. April 1) and predisposed to an onset of MD that began in June, that case would fall outside our assessment period. So, our overall effect estimates for our ERS are likely somewhat underestimated.

We were sufficiently concerned about this possible loss of power to perform sensitivity analyses using the same linear model employed in Table 2 to examine the impact on risk for MD in our 6-month follow-phase of our cohort for our ERS assessed by month 1, 2, 3, 4, 5 and 6 months prior to our September 1, 2010 cut-off. As seen in Supplementary Table 6, the effects of the ERS on MD risk were highest within 2 months of the January 9, 2010 cut-off but declined only slightly out to 6 months suggesting that we experienced only a modest loss of “signal” here.

Third, given our goal of developing an aggregate SLE measure in a population-based cohort, we did not model the necessary conditionality of some of our categories of SLEs condition. For example, individuals with no spouses or children, could not experience any of our assessed spouse and child-related SLEs, respectively. This introduces a conservative bias into our aggregate estimates of the impact of our ERS measure.

Fourth, by design, our current ERS is “tuned” to predict MD. We hope in the future, to apply this method to other psychiatric conditions such as Alcohol Use Disorder. For that condition, we would need to repeat our split-half method and would likely have different weightings than were optimal for MD.

Fifth, some of our registry-based SLEs might substantially overlap with one another. To evaluate the magnitude of this problem, we examined, in Supplementary Tables 7–11, tetrachoric correlations between our individual SLEs in our entire sample for five groups: events to the individual, disorder diagnoses in the individual, and events to spouse, parents and siblings, and child. Considering correlations >0.50 as “high,” across all these tables, we had 271 correlations of which 13 (4.8%) met that standard. Examples would include for individuals “accidents and fractures,” for spouses “Hemorrhagic Stroke and Subarachnoid Hemorrhage” and children “severe psychiatric illness and suicidal behavior.” These results suggest that content overlap among our individual SLEs is not a major problem in the interpretation of our findings.

Sixth, given that our weighting of the individual SLEs was empirical, it is appropriate to ask how reproducible these weights were. We therefore compared the weights generated in our training and testing split-halves in Supplementary Figure 2. They were virtually identical to all SLEs as might be predicted given our large sample size.

Seventh, do the empirical weightings we developed for our events actually add to the predictive power of our ERS? To address this question, we compared the predictive power of our weighted model seen in Table 2 with that obtained from an unweighted sum of events. As assessed by the variance accounted for, the weighted model’s predictive power was 80% greater than the unweighted model.

Finally, we do not have measures of self-report onsets for MD but rather need to rely on first registrations in the Swedish medical registries. Access to medical care is very widely available in Sweden and the population prevalence of treated MD nationally from those born from 1970 to 1990, equals 20.0 in females and 10.8% in males which suggests that we are not substantially under-ascertaining this disorder (Kendler, Ohlsson, Lichtenstein, Sundquist, & Sundquist, 2018). However, there is likely a variable delay from MD onset to seeking health care which is more likely to attenuate than exaggerate the temporal associations we have observed between the stressors and depressive onsets.

## Conclusions

We report the development of an ERS obtained from registry data and tested it in the prediction of MD. This method has several important advantages in SLE assessment compared to questionnaire or interview-based measures, including objectivity in the assessment, lack of dependence on recall biases and inaccuracies in dating, and scalability to large samples. It is, however, not without important limitations, especially the inability to assess certain important areas of adversity and the lack of precise timing of the onsets of depressive episodes. We here show that the association of our ERS with MD episodes is robust and we replicate prior findings including increased depressive risk in women versus men and those at high versus low genetic risk. We also show the effects on depressive risk of our ERS to be largely but not entirely causal. This and similar registry-based methods for SLE assessment are likely to be useful in developing large-sample general population-based models of how environmental adversities and genetic vulnerability contribute to the onset and recurrence of a number of major psychiatric and substance use disorders.

## Supporting information

Kendler et al. supplementary material Kendler et al. supplementary material

## Data Availability

Kristina Sundquist had full access to all the data in the study and takes responsibility for the integrity of the data and the accuracy of the data analysis.

## Supplementary material

The supplementary material for this article can be found at http://doi.org/10.1017/S0033291725000583.
