# Supplementary material for: The development of an environmental risk score using Swedish National Registers and its impact on subsequent episodes of major depression
Source: Psychol Med. 2025 Mar 7;55:e74. doi: 10.1017/S0033291725000583 (PMC12080639; doi:10.1017/S0033291725000583)

Appendix

Table 1 -Sources for Our Dataset

Total Population Register, containing information about year of birth, sex, family and marital status; Multi-Generation Register, linking individuals born after 1932 to their parents, the Hospital Discharge Register, containing hospitalizations for Swedish inhabitants from 1964-2018; Outpatient Care Register, containing information from all outpatient clinics from 2001 to 2018; and regional Primary Health Care Registers data from Blekinge (2009 - 2018), Dalarna (2005 - 2018), Värmland (2005 - 2018), Kalmar Län (2007 - 2018), Sörmland (1997 - 201), Uppsala Län (2005 - 2018), Västernorrland (2010 - 2018) Norrbotten Län (2001 - 2018), Gävleborg (2010 - 2018), Gotland (2011 – 2018), Halland (2007 - 2018), Jönköpings Län (2008 - 2018), Kronoberg (2006 - 2018), Skåne (1989 - 2018), Västerbotten (1992- 2018), Östergötland (1990 - 2018), Stockholms Län (2003 - 2018), Västmanland (2014 to 2018), and Västra Götaland (2000 - 2018). The time periods varied due to the regions’ different timing of digitalizing of the patient records. In addition, we used the Crime Register that included national complete data on all convictions in lower court from 1973-2018; and the Mortality Register with dates and causes of death from 1952 until 2018.

Table 2 List of events Occurring to the Individual included in the environmental risk score (ERS)

|  | ICD-10 |
| --- | --- |
| Fire or Explosion | X00–X08, T20–T25, T31 |
| Transport accidents | V01–V99 |
| Exposure to toxic substance/medical complications | T15–T98 (except: T20–T25, T31 T74) |
| Physical assault | X92, Y01–Y04 |
| Assault with a weapon | X93–Y00 |
| Sexual assault | T74.2, Y05 |
| Violent victimization (not included elsewhere) | X85–Y04 Y06-Y09 |
| Pregnancy-related trauma | O60–O75 |
| Accidents | W00-W19 Slipping, tripping, stumbling and falls, W20-W49 Exposure to inanimate mechanical forces, W50-W64 Exposure to animate mechanical forces, W65-W74 Accidental non-transport drowning and submersion, W85-W99 Exposure to electric current, radiation and extreme air temperature or pressure |
| War or terror | Y35, Y36, Y37, Y38 |
| Other nonviolent accidents  (Contact with heat and hot substances, Exposure to forces of nature, Overexertion and strenuous or repetitive movements, Accidental exposure to other specified factors) | X10-X19.  X30-X39  X50-X50  X52-X58 |
| Note on Medical Misadventure | Y40 - Y84 |
| Fractures | S12, S22, S32, S42, S52, S62, S72, S82, S92, T02, T08, T10,  T12, T14.2 |
| Diseases of the blood and blood-forming organs and certain disorders involving the immune mechanism. | D60  D61.1  D86 |
| Endocrine, nutritional and metabolic diseases (Diabetes) | E10, E11, E14 |
| Diseases of the nervous system | G50, G51, G53, G61.0, G62.0, G62.2, G63 |
| Diseases of the circulatory system | I00, I01, I05, I06, I07,  I08, I09, I20, I21, I22,  I23, I24, I25, I26, I30, I31, I32, I33, I38, I39, I40, I41, I48, I71, I72, I74, I77, I81, I82, I87.0 |
| Diseases of the Respiratory System | J43, J44, J45, J80, J81,  J86, J93, J94,  J98.2, J99.0  J99.1 |
| Diseases of the digestive system | K22.1, K22.2, K22.3, K29.0, K50, K51, K55, K60, K61, K74.3, K74.4, K75, K76, K81, K82, K85, K86.1, K86.8 |
| Diseases of the skin and subcutaneous tissue | L05, L10.0, L12.0,  L40.1, L40.5, L93 |
| Diseases of the musculoskeletal system and connective tissue | M05, M06, M07, M13, M15, M16, M17, M30.3, M31, M32, M33.2, M33.9, M34, M35.0, M35.1, M35.2, M35.3, M35.4, M35.5, M35.6, M36, M45, M50-M52, M80, M84, M86, M87, M88, M96 |
| Diseases of the genitourinary system | N00, N01, N03, N17,  N18, N19, N26, N28,  N80, N94, N99 |
| Cancer | All malign tumors (C0-C9) except ​Malignant tumors of the eye, brain and other parts of the central nervous system, C69-C72 |
| Conviction of violent crime (VC), white collar crime (WCC) and property crime (PC) | VC – (aggravated) assault, illegal threats, intimidation and illegal coercion, threats or violence against a police officer, (aggravated) robbery, murder, manslaughter or filicide, kidnapping, arson, sexual crimes (excluding prostitution and the buying of sexual services but including child pornography); WCCB – fraud, forgery and dishonesty, and embezzlement; and PCB – theft, vandalism, vandalism causing danger to the public and trespassing. |

Table 3 – List of events Occurring to the spouse, parents, and full siblings* included in the environmental risk score (ERS) (not counted if parents or siblings are over the age of 80 at the time of the diagnosis).

| **Diagnosis** | **ICD codes** |
| --- | --- |
| Septic shock | A40, A41 |
| Heart attack | I21 |
| Thrombotic stroke | I63 |
| Hemorrhagic stroke | I61 |
| Subarachnoid hemorrhage | I60 |
| Severe cancer | C16, C34, C15, C22, C71, C25, C45 |
| Parkinson’s | G20.9 |
| Dementia | F00 – F03 |
| ALS | G12.2 |
| Suicide | X6, X7, X80-X84 from the Mortality register only |

Table 4 – List of severe events occurring to children (if under age 18 at the time of the diagnosis) included in the environmental risk score (ERS).

| **Diagnosis** | **ICD codes** |
| --- | --- |
| Diagnosis of any cancer | Any cancer |
| Psychiatric disease or psychosis: | F20, F31 |
| External causes of morbidity and mortality, including suicidal behavior self-harm and/or suicide attempt. | V01-Y98, X60-X84, Y10-Y34 |
| Septic shock | P36, A40, A41 |
| Inflammatory bowel disease | K50, K51 |
| Diabetes type 1 | E10 |
| Criminal convictions | Any conviction in lower court if under age 18. Our definition of CB (excluding milder event like traffic and drug use) if over age 18 |

Table 5 – Logistic Regression Analysis on the Percentage Scale of the impact of our ERS and Prior MD Episodes on Risk for MD During the 6-mont Follow-up Period – Parameters and 95% Cis

|  | Percent scale, so that the MD ERS is on a scale 0 to 100 and the OR represent an increase in 1%.  So, different from the scale we used in the linear models. |
| --- | --- |
| MD ERS | 1.34 (1.32 - 1.37) |
| MD before | 43.11 (41.15 -45.15) |
| MD ERS × MD before | 0.81 (0.79 - 0.83) |

Table 6 - Regression Coefficient from our Linear Model for our ERS in the Prediction of MD in the Six Months After 9/1/2010 by ERS Exposure Period Relative to the 9/1/10 cut-off*

| **Exposure period before 9/1/10** | **Coefficient** |
| --- | --- |
| 0 to 1 month prior | 1.21 (1.13, 1.28) |
| 1 to 2 month prior | 1.21 (1.13, 1.29) |
| 2 to 3 month prior | 1.19 (1.11, 1.28) |
| 3 to 4 month prior | 1.07 (0.99, 1.14) |
| 4 to 5 month prior | 0.98 (0.90, 1.05) |
| 5 to 6 month prior | 1.00 (0.92, 1.07) |
| That is, 0-1 month prior would be 8/1 to 8/31/2010, etc | |

***Tables 7 – Tetrachoric Correlations Between Individual Stressful Life events – Events to the individual. Correlations > 0.50 highlighted***

FE = Fire or Explosion

TX = Exposure to Toxic Substance

TAC = Transport Accidents

PA = Physical Assault

WA = Assault with a Weapon

OVV = Other Violent Victimization

PRT = Pregnancy Related Trauma

ACC = Accidents

HE = Heat and hot substances

MM = Note on Medical Misadventure

FNE = Forces of nature

OR = Overexertion and strenuous

AE = Accidental Exposure

FRC = Fracture

SXA = Sexual Assault

CB = Criminal Conviction

|  | **FE** | **TX** | **TAC** | **PA** | **WA** | **OVV** | **PRT** | **ACC** | **HE** | **MM** | **FNE** | **OR** | **AE** | **FRC** | **SXA** | **CB** |
| --- | --- | --- | --- | --- | --- | --- | --- | --- | --- | --- | --- | --- | --- | --- | --- | --- |
| **FE** | 1 | 0.31 | 0.16 | 0.17 | 0.35 | 0.25 | 0.06 | 0.26 | 0.93 | 0.16 | 0.55 | 0.12 | 0.39 | 0.12 | 0.27 | 0.14 |
| **TX** | 0.31 | 1 | 0.3 | 0.22 | 0.27 | 0.27 | -0.03 | 0.5 | 0.23 | 0.67 | 0.49 | 0.27 | 0.47 | 0.34 | 0.23 | 0.15 |
| **TAC** | 0.16 | 0.3 | 1 | 0.17 | 0.12 | 0.16 | -0.05 | 0.31 | 0.12 | 0.12 | 0.14 | 0.13 | 0.15 | 0.61 | 0.11 | 0.16 |
| **PA** | 0.17 | 0.22 | 0.17 | 1 | 0.59 | 0.66 | -0.07 | 0.31 | 0.22 | 0.15 | 0.3 | 0.13 | 0.22 | 0.35 | 0.4 | 0.36 |
| **WA** | 0.35 | 0.27 | 0.12 | 0.59 | 1 | 0.54 | 0.02 | 0.3 | 0.37 | 0.17 | 0.41 | 0.15 | 0.28 | 0.28 | 0.45 | 0.41 |
| **OVV** | 0.25 | 0.27 | 0.16 | 0.66 | 0.54 | 1 | -0.06 | 0.29 | 0.17 | 0.11 | 0.28 | 0.12 | 0.24 | 0.3 | 0.59 | 0.36 |
| **PRT** | 0.06 | -0.03 | -0.05 | -0.07 | 0.02 | -0.06 | 1 | -0.15 | 0.1 | 0.07 | 0.11 | -0.03 | 0.01 | -0.17 | 0.07 | -0.04 |
| **ACC** | 0.26 | 0.5 | 0.31 | 0.31 | 0.3 | 0.29 | -0.15 | 1 | 0.23 | 0.17 | 0.22 | 0.34 | 0.28 | 0.85 | 0.12 | 0.15 |
| **HE** | 0.93 | 0.23 | 0.12 | 0.22 | 0.37 | 0.17 | 0.1 | 0.23 | 1 | 0.13 | 0.37 | 0.12 | 0.32 | 0.09 | 0.31 | 0.11 |
| **MM** | 0.16 | 0.67 | 0.12 | 0.15 | 0.17 | 0.11 | 0.07 | 0.17 | 0.13 | 1 | 0.23 | 0.08 | 0.23 | 0.17 | 0.16 | 0.03 |
| **FNE** | 0.55 | 0.49 | 0.14 | 0.3 | 0.41 | 0.28 | 0.11 | 0.22 | 0.37 | 0.23 | 1 | 0.29 | 0.35 | 0.15 | 0.45 | 0.19 |
| **OR** | 0.12 | 0.27 | 0.13 | 0.13 | 0.15 | 0.12 | -0.03 | 0.34 | 0.12 | 0.08 | 0.29 | 1 | 0.29 | 0.43 | 0.21 | 0.08 |
| **AE** | 0.39 | 0.47 | 0.15 | 0.22 | 0.28 | 0.24 | 0.01 | 0.28 | 0.32 | 0.23 | 0.35 | 0.29 | 1 | 0.29 | 0.22 | 0.14 |
| **FRC** | 0.12 | 0.34 | 0.61 | 0.35 | 0.28 | 0.3 | -0.17 | 0.85 | 0.09 | 0.17 | 0.15 | 0.43 | 0.29 | 1 | 0.09 | 0.11 |
| **SXA** | 0.27 | 0.23 | 0.11 | 0.4 | 0.45 | 0.59 | 0.07 | 0.12 | 0.31 | 0.16 | 0.45 | 0.21 | 0.22 | 0.09 | 1 | 0.27 |
| **CB** | 0.14 | 0.15 | 0.16 | 0.36 | 0.41 | 0.36 | -0.04 | 0.15 | 0.11 | 0.03 | 0.19 | 0.08 | 0.14 | 0.11 | 0.27 | 1 |

**Table 8 Tetrachoric Correlations Between Individual Stressful Life events – Disorders in the individual. Correlations > 0.50 highlighted**

DBO = Diseases of the Blood

DM = Any Diabetes

DNS = Diseases of the Nervous System

DCS = Diseases of the Circulatory System

DRS = Diseases of the Respiratory System

DDS = Diseases of the Digestive System

DST = Diseases of the Skin

DMS = Diseases of the Musculoskeletal System

DGS = Diseases of the Genitourinary System

DGSf = Diseases of the Female Genitourinary System

CAN = Cancer

|  | **DBO** | **DM** | **DNS** | **DCS** | **DRS** | **DDS** | **DST** | **DMS** | **DGS** | **DGSf** | **CAN** |
| --- | --- | --- | --- | --- | --- | --- | --- | --- | --- | --- | --- |
| **DBO** | 1 | 0.14 | 0.24 | 0.19 | 0.19 | 0.18 | 0.19 | 0.2 | 0.3 | -0.03 | 0.33 |
| **DM** | 0.14 | 1 | 0.17 | 0.34 | 0.2 | 0.18 | 0.09 | 0.15 | 0.24 | -0.06 | 0.2 |
| **DNS** | 0.24 | 0.17 | 1 | 0.17 | 0.11 | 0.1 | -0.02 | 0.1 | 0.16 | 0 | 0.14 |
| **DCS** | 0.19 | 0.34 | 0.17 | 1 | 0.29 | 0.22 | 0.07 | 0.18 | 0.33 | -0.09 | 0.28 |
| **DRS** | 0.19 | 0.2 | 0.11 | 0.29 | 1 | 0.13 | 0.1 | 0.13 | 0.22 | 0.07 | 0.21 |
| **DDS** | 0.18 | 0.18 | 0.1 | 0.22 | 0.13 | 1 | 0.33 | 0.08 | 0.23 | 0.08 | 0.22 |
| **DST** | 0.19 | 0.09 | -0.02 | 0.07 | 0.1 | 0.33 | 1 | 0.39 | 0.07 | 0.06 | 0.02 |
| **DMS** | 0.2 | 0.15 | 0.1 | 0.18 | 0.13 | 0.08 | 0.39 | 1 | 0.18 | -0.01 | 0.09 |
| **DGS** | 0.3 | 0.24 | 0.16 | 0.33 | 0.22 | 0.23 | 0.07 | 0.18 | 1 | 0.06 | 0.28 |
| **DGSf** | -0.03 | -0.06 | 0 | -0.09 | 0.07 | 0.08 | 0.06 | -0.01 | 0.06 | 1 | 0.02 |
| **CAN** | 0.33 | 0.2 | 0.14 | 0.28 | 0.21 | 0.22 | 0.02 | 0.09 | 0.28 | 0.02 | 1 |

***Table 9*** ***Tetrachoric Correlations Between Individual Stressful Life events – Events in the Spouse. Correlations > 0.50 highlighted***

|  | **Cancer** | **Heath Attack** | **Hemorrhagic Stroke** | **Thrombotic Stroke** | **Subarachnoid Hemorrhage** | **Septic shock** | **ALS** | **Parkinson’s** | **Death** |
| --- | --- | --- | --- | --- | --- | --- | --- | --- | --- |
| **Cancer** | 1 | 0.29 | 0.32 | 0.28 | 0.21 | 0.35 | 0.26 | 0.13 | -0.09 |
| **Heath Attack** | 0.29 | 1 | 0.3 | 0.39 | 0.29 | 0.38 | 0.22 | 0.24 | -0.18 |
| **Hemorrhagic Stroke** | 0.32 | 0.3 | 1 | 0.52 | 0.63 | 0.27 | 0.31 | 0.18 | -0.05 |
| **Thrombotic Stroke** | 0.28 | 0.39 | 0.52 | 1 | 0.44 | 0.3 | 0.25 | 0.24 | -0.19 |
| **Subarachnoid Hemorrhage** | 0.21 | 0.29 | 0.63 | 0.44 | 1 | 0.35 | 0.38 | 0.2 | 0 |
| **Septic shock** | 0.35 | 0.38 | 0.27 | 0.3 | 0.35 | 1 | 0.26 | 0.27 | -0.12 |
| **ALS** | 0.26 | 0.22 | 0.31 | 0.25 | 0.38 | 0.26 | 1 | 0.24 | 0.04 |
| **Parkinson’s** | 0.13 | 0.24 | 0.18 | 0.24 | 0.2 | 0.27 | 0.24 | 1 | -0.18 |
| **Death** | -0.09 | -0.18 | -0.05 | -0.19 | 0 | -0.12 | 0.04 | -0.18 | 1 |

***Table 10 Tetrachoric Correlations Between Individual Stressful Life events – Events in the Parents and Siblings. Correlations > 0.50 highlighted***

|  | **Parental Death** | **Parental Suicide** | **Severe disease in parents** | **Death of full sibling** | **Suicide in full sibling** | **Severe disease in full sibling** |
| --- | --- | --- | --- | --- | --- | --- |
| **Parental Death** | 1 | 0.86 | 0.55 | 0.03 | 0.07 | 0.05 |
| **Parental Suicide** | 0.86 | 1 | 0.12 | 0.11 | 0.18 | -0.01 |
| **Severe disease in parents** | 0.55 | 0.12 | 1 | -0.02 | 0.06 | -0.02 |
| **Death of full sibling** | 0.03 | 0.11 | -0.02 | 1 | 0.9 | 0.53 |
| **Suicide in full sibling** | 0.07 | 0.18 | 0.06 | 0.9 | 1 | 0.13 |
| **Severe disease in full sibling** | 0.05 | -0.01 | -0.02 | 0.53 | 0.13 | 1 |

***Table 11 -Tetrachoric Correlations Between Individual Stressful Life events – Events in the Child Spouse. Correlations > 0.50 highlighted***

|  | **Type 1 diabetes** | **IBD** | **Severe Psychiatric**  **Diagnosis** | **Septic shock** | **Suicidal Behavior** | **Accident** | **Cancer** | **Criminal behavior** | **Death** | **Suicide** |
| --- | --- | --- | --- | --- | --- | --- | --- | --- | --- | --- |
| Type 1 diabetes | 1 | 0.21 | 0.24 | 0.24 | 0.19 | 0.2 | 0.21 | 0.01 | 0.16 | 0.2 |
| IBD | 0.21 | 1 | 0.32 | 0.31 | 0.25 | 0.18 | 0.28 | 0.12 | 0.07 | 0.26 |
| Severe Psychiatric Diagnosis | 0.24 | 0.32 | 1 | 0.35 | 0.65 | 0.2 | 0.32 | 0.26 | 0.24 | 0.3 |
| Septic shock | 0.24 | 0.31 | 0.35 | 1 | 0.29 | 0.2 | 0.88 | 0.1 | 0.55 | 0.3 |
| Suicidal Behavior | 0.19 | 0.25 | 0.65 | 0.29 | 1 | 0.25 | 0.25 | 0.38 | 0.33 | 0.5 |
| Accident | 0.2 | 0.18 | 0.2 | 0.2 | 0.25 | 1 | 0.17 | 0.17 | 0.07 | 0.14 |
| Cancer | 0.21 | 0.28 | 0.32 | 0.88 | 0.25 | 0.17 | 1 | 0.15 | 0.53 | 0.27 |
| Criminal behavior | 0.01 | 0.12 | 0.26 | 0.1 | 0.38 | 0.17 | 0.15 | 1 | 0.54 | 0.48 |
| Death | 0.16 | 0.07 | 0.24 | 0.55 | 0.33 | 0.07 | 0.53 | 0.54 | 1 | 0.88 |
| Suicide | 0.2 | 0.26 | 0.3 | 0.3 | 0.5 | 0.14 | 0.27 | 0.48 | 0.88 | 1 |

Figure 1 – Sex Differences in Risk for MD Across our Individual SLEs


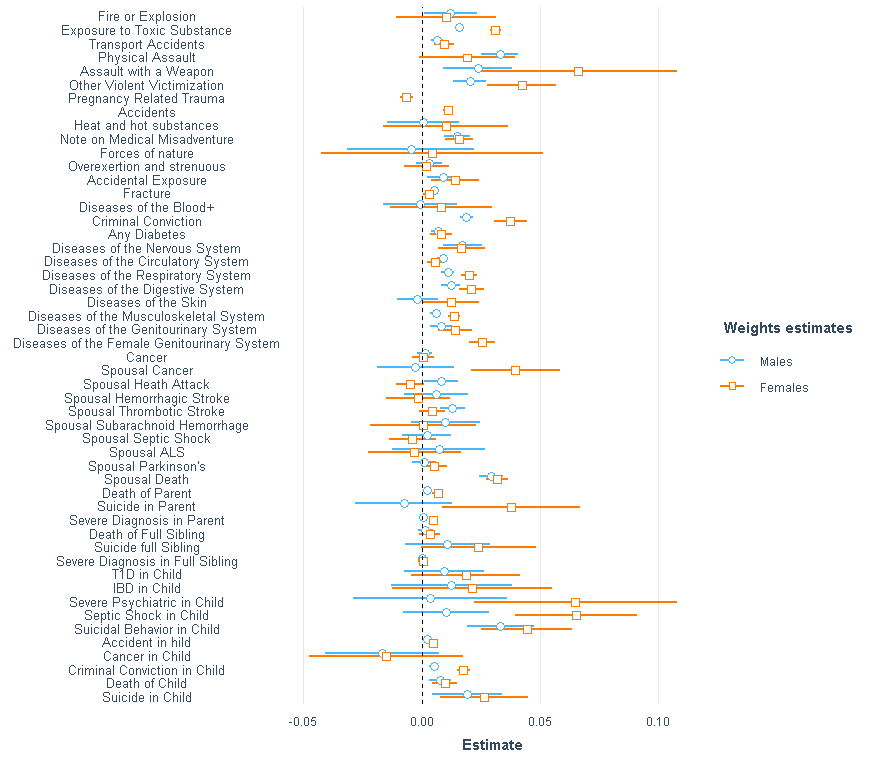


Figure 2 – Weight Estimates for our SLEs in our Training and Testing Split-Halves


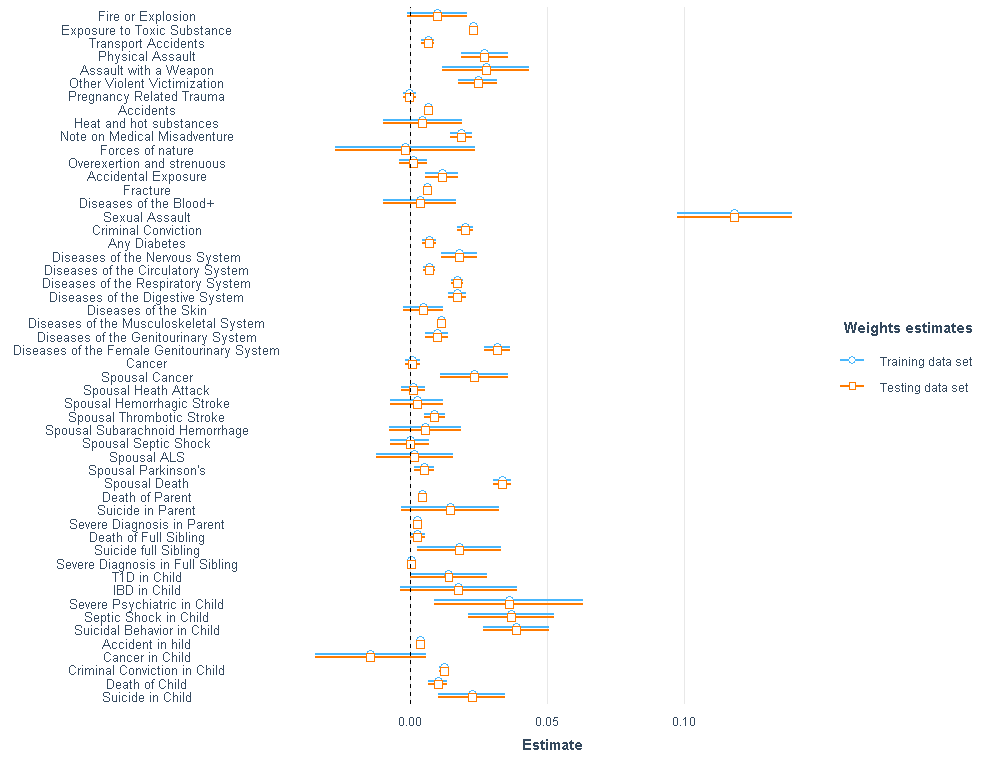

Supplement: Kendler et al. supplementary material [file S0033291725000583sup001.docx]
